# Sequential Filtering Processes Shape Feature Detection in Crickets: A Framework for Song Pattern Recognition

**DOI:** 10.3389/fphys.2016.00046

**Published:** 2016-02-25

**Authors:** Berthold G. Hedwig

**Affiliations:** Department of Zoology, University of CambridgeCambridge, UK

**Keywords:** feature detection, calling song, onset activity, reciprocal inhibition, delay line, coincidence detector, post-inhibitory rebound, modulation

## Abstract

Intraspecific acoustic communication requires filtering processes and feature detectors in the auditory pathway of the receiver for the recognition of species-specific signals. Insects like acoustically communicating crickets allow describing and analysing the mechanisms underlying auditory processing at the behavioral and neural level. Female crickets approach male calling song, their phonotactic behavior is tuned to the characteristic features of the song, such as the carrier frequency and the temporal pattern of sound pulses. Data from behavioral experiments and from neural recordings at different stages of processing in the auditory pathway lead to a concept of serially arranged filtering mechanisms. These encompass a filter for the carrier frequency at the level of the hearing organ, and the pulse duration through phasic onset responses of afferents and reciprocal inhibition of thoracic interneurons. Further, processing by a delay line and coincidence detector circuit in the brain leads to feature detecting neurons that specifically respond to the species-specific pulse rate, and match the characteristics of the phonotactic response. This same circuit may also control the response to the species-specific chirp pattern. Based on these serial filters and the feature detecting mechanism, female phonotactic behavior is shaped and tuned to the characteristic properties of male calling song.

## Introduction

In many species of insects, intraspecific signaling systems have evolved to allow mate attraction over long distances, including systems based on sex pheromones in moths and butterflies (Jacobsen, 1972), light patterns in fireflies (Carlson and Copeland, 1985; Lewis and Cratsley, 2008) and acoustic signals in orthoptera and hemiptera (Busnel, 1963; Alexander, 1967; Hedwig, 2014). These specialized communication systems are shaped by evolution so that both the signal generation and recognition processes are selective to a species-specific pattern. As intraspecific communication is crucial for the animals' mating success it requires reliable performance at the sender and the receiver side. The species-specific signals emitted by a sender require matched detection and recognition mechanisms by the receiver. Signal generation and signal recognition processes in insects are implemented in rather simple nervous systems and therefore provide a chance to unravel the underlying neural mechanisms at a cellular level.

The acoustic behavior of crickets is an established model system to analyse the neurobiological basis of auditory processing. Females approach singing males by phonotaxis, using only acoustic cues for pattern recognition and subsequent orientation. Considerable research in this field is aimed to understand how the temporal pattern of male calling song is recognized by the female nervous system (Popov et al., 1974; Hoy, 1978; Huber, 1978). As outlined in different hypothesis (review by Kostarakos and Hedwig, 2015), for pattern recognition to occur single neurons or networks of neurons should selectively respond to the species-specific characteristics of a signaling pattern. These are known as “feature detector” neurons or networks (Bullock, 1961; Hoy, 1978). Revealing the cellular and network mechanisms that lead to the selectivity of these neurons provides the opportunity to understand how a sensory system has been shaped during evolution to specifically process behaviorally relevant stimuli (Konishi, 1991).

In crickets, phonotaxis toward a species-specific calling song requires that its salient features are reliably detected, processed, and transformed into an appropriate motor response by the nervous system. Here a framework is outlined that calling song pattern recognition is organized in a set of serial filters, with each filter selectively responding to a particular characteristic of the song. At each level of auditory processing, a different feature of the calling song is extracted from the overall original signal leading eventually to the very specific activity of feature-detecting neurons in the brain.

This outline for song pattern recognition is mainly based on data in the sister species of *G. bimaculatus* and *G. campestris*, which have similar sound patterns and auditory preferences (Thorson et al., 1982). It however should provide a framework for different species of crickets as well as for the processing of communication signals in other specialized sensory pathways. Note, that data regarding auditory thresholds and tuning may slightly vary in papers cited, as different experimental procedures and recording methods were used.

## The male calling song and the female auditory challenge

**Figure 1:**
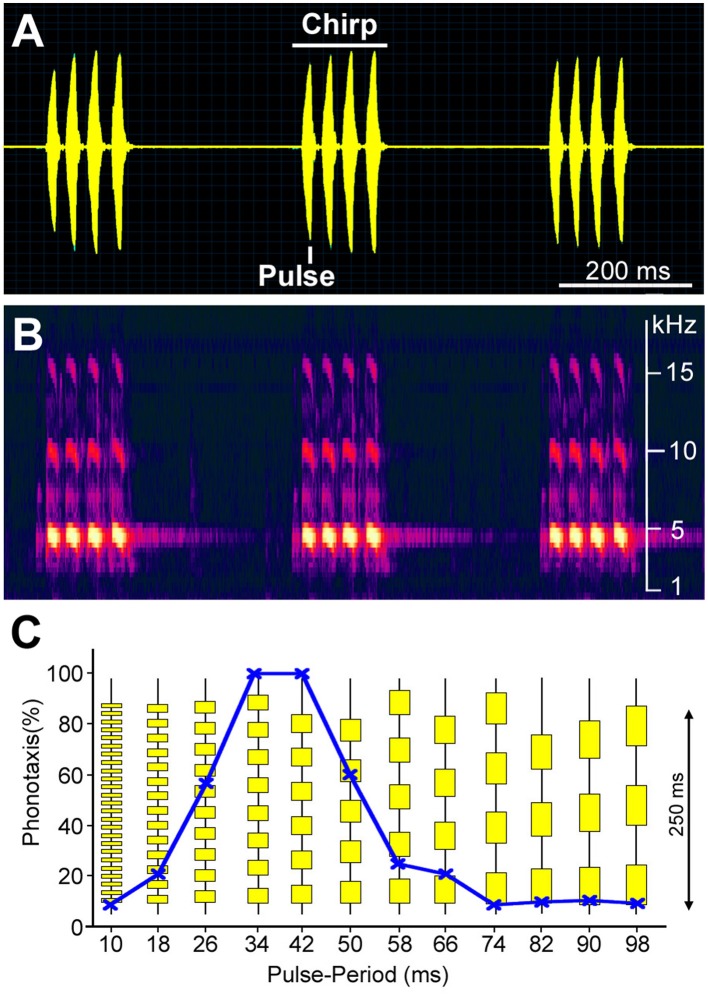
**Sound pattern of a male ***G. bimaculatus*** calling song in the temporal (A) and frequency domain (B)**. Chirps with four sound pulses are repeated at a rate of 3 per second. The main carrier frequency of the sound is around 4.8 kHz, with corresponding higher harmonics. **(C)** Tuning of female phonotactic behavior toward different temporal patterns of sound pulses as revealed in trackball experiments (Hedwig and Poulet, 2005). Test patterns of chirps with different pulse durations and intervals are vertically arranged. Each pattern was presented for 30 s from the left and right hand side while the cricket's phonotactic steering toward the stimulus was recorded.

Only male crickets sing, which they achieve by the rhythmic opening and closing of their elevated front wings, with sound generated only on the closing movements. Males of different species produce different species-specific patterns of sound pulses in the contexts of mate attraction, courtship and rivalry behavior (Alexander, 1962; Otte, 1992). During calling song in *Gryllus bimaculatus* (Figures 1A,B), sound pulses are 15–20 ms long, separated by 15–20 ms silent intervals; and grouped into chirps of 3–5 pulses, which are repeated at a rate of 3–4 chirps/s (Doherty, 1985). Sound pulses rise to a maximum intensity of about 100 dB Sound Pressure Level (SPL) within a few milliseconds, and have a carrier frequency of around 4.8 kHz. Thus, the typical calling song of *G. bimaculatus* is characterized by four features: carrier frequency, pulse duration, pulse repetition rate, and chirp structure, which is given by the number of pulses per chirp and the interchirp interval. In male-male interactions, variable short rivalry songs are generated with chirps comprising 6–12 pulses. When courting a female, males generate single sound pulses at the chirp rate of the calling song, but with carrier frequencies of 11–16 kHz (Libersat et al., 1994). As compared to the more episodic courtship and rivalry song, which are accompanied by other sensory signals, e.g., antennal contact, and emitted in close male-female and male-male encounters, the stereotypic calling song is a long distance communication signal which may be emitted continuously for many hours to attract females.

Sexually-receptive females walk or fly toward a singing male, using only the male's acoustic cues as guidance for their phonotactic orientation. The tuning of their phonotactic behavior matches the temporal pattern of the male calling song (Figure 1C). They prefer pulse patterns similar to calling song, and are not attracted by short pulses repeated at a high repetition rate or by long pulses repeated at a lower rate (Thorson et al., 1982; Hedwig, 2006). Female *G. bimaculatus* and *G. campestris* therefore show a band-pass tuning of their phonotactic behavior based on pulse duration and pulse interval. Sound pulses also need to be at the species' typical carrier frequency to be attractive.

## Serial filter processes underlying calling song recognition

**Figure 2:**
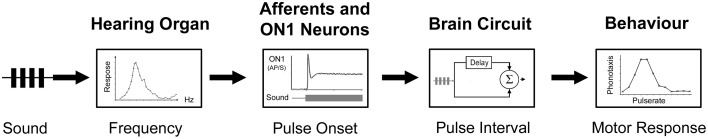
**Schematic diagram of serial filter processes in the cricket auditory pathway**. At each stage, the response specificity is enhanced. Left to right: frequency tuning of the tympanic membrane response is based on biophysical filter mechanisms in the hearing organ; the detection of sound pulses is supported by the phasic-tonic response properties of auditory afferents and by reciprocal inhibition at the level of thoracic interneurons; the pulse rate is processed in the brain by a delay line coincidence detecting circuit, driving feature detector neurons that preferentially respond to the species-specific pulse rate. In addition, the feature detection circuit may modulate a reactive non-selective steering pathway at the time scale of the chirp rate (not shown). Finally, phonotactic walking behavior is tuned to sound pulses of the calling song carrier frequency presented at the species-specific pulse duration and pulse interval.

Current data indicate that the properties of the peripheral and central auditory pathway are specifically adapted to process the male calling song. This process is organized in a set of serially arranged filter mechanisms (Figure 2) that finally leads to a highly selective response of feature detecting brain neurons and the tuned phonotactic behavior. First, at the level of the hearing organ a peripheral filter is selective for the song carrier frequency; second is a neural filter mechanism of phasic afferent and interneuronal activity which enhances the response to the onset of sound pulses; and third is a neural network in the brain with a delay line and coincidence detector feeding into feature detecting neurons which are tuned to a specific pulse repetition rate. The detection of the species-specific pulse rate may also control the phonotactic steering responses to the chirp pattern. All these filters together contribute to and shape the band-pass tuning of the cricket phonotactic orientation behavior.

### Processing of the calling song carrier frequency

**Figure 3:**
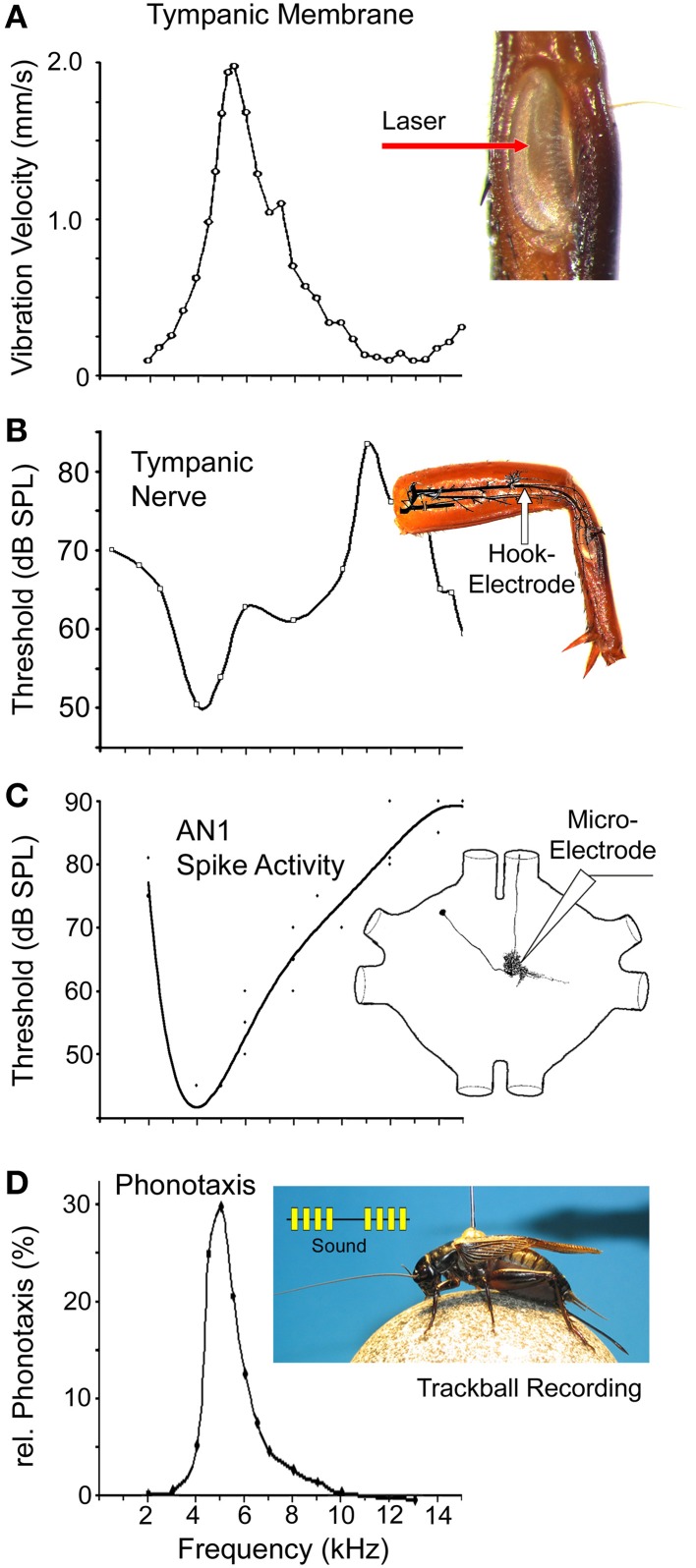
**Frequency tuning at different processing stages in the auditory pathway of field crickets. (A)** Vibration velocity amplitude of the posterior tympanic membrane in *G. bimaculatus* as measured with laser vibrometry at a constant sound amplitude of 94 dB SPL while acoustic spiracles were blocked. Redrawn from Larsen (1981). Inset shows the posterior tympanal membrane in the tibia. **(B)** Frequency tuning of the auditory organ. Threshold of the summed activity of the tympanal nerve in *G. campestris*. Redrawn on a linear frequency scale, after Nocke (1972). Inset shows the recording site of the auditory nerve in the femur. **(C)** Threshold for frequency tuning of AN1 spike activity in *G. bimaculatus*. Data pooled and redrawn from Rheinlaender et al. (1976); Schildberger et al. (1989), and Horseman and Huber (1994). Inset shows structure of AN1. **(D)** Frequency tuning of phonotactic behavior in *G. bimaculatus* based on auditory steering responses of tethered females walking on a trackball system. Calling song pattern presented at 75 dB SPL with varying carrier frequency. Data based on seven tests. Inset shows sound pattern and cricket on a trackball.

In all auditory systems, frequency processing starts at the biophysical level. Due to the mechanical filtering properties of the peripheral transduction mechanism, frequency components are separated and forwarded to spatially distinct structures of the hearing organ in a frequency-specific way as revealed in the ears of moths, locusts, and cicada (Windmill et al., 2005, 2007; Sueur et al., 2006). Oscillations of these structures then drive the activity of afferent neurons in the hearing organs. In the field cricket *G. bimaculatus* the carrier frequencies of calling songs cover a range of 4.3–5.2 kHz (Kostarakos et al., 2009), and courtship songs are in the range of 11–16 kHz (Libersat et al., 1994). The biophysics of the peripheral auditory system allows for selective responses at the level of the auditory afferents to these low and high frequency components of the communication signals (Oldfield et al., 1986). The afferent activity is then carried forward to the central nervous system where it sets the limits for the subsequent frequency tuning of central interneurons, and finally the categorical phonotactic responses (Wyttenbach et al., 1996; Figures 2, 3).

#### The peripheral auditory system: tuning of tympanic membrane vibrations

The peripheral auditory system in crickets is characterized by a small frontal and a large posterior tympanic membrane, which are located on the tibia of each front leg. The hearing organ is positioned behind the posterior tympanum where a row of 40–60 auditory afferents is arranged in a structure known as the crista acustica. The organ is attached to the auditory trachea (Michel, 1974), which extends from the front tibia to the first thoracic segment where it ends with a lateral opening at the auditory spiracle (Nocke, 1972; Huber and Thorson, 1985). Sound enters the auditory system via the spiracles of the auditory trachea, and also via the posterior tympanic membrane in the tibia. For directional coding, the efficiency of the different sound pathways depends on the carrier frequency and the angle of incidence (Michelsen et al., 1994; Seagraves and Hedwig, 2014). The peripheral auditory pathway also provides the essential step of frequency filtering. Movements of the posterior tympanal membrane are necessary for hearing in crickets (Kleindienst et al., 1983) and mirror the frequency tuning of the auditory system. Laser vibrometry measurements of the mechanical oscillations of the posterior tympanic membrane in *G. bimaculatus* (Larsen, 1981) revealed the best response at 5.3 kHz (Figure 3A); the velocity response drops toward 2 kHz and decreases toward 14 kHz. Like in other species of crickets (Johnstone et al., 1970; Paton et al., 1977), these data indicate that the mechanical response of the peripheral auditory system matches the calling song carrier frequency (Figure 1B). Since these early measurements, the oscillation properties of tympanic membranes in field cricket have not been studied any further; using more recent laser technology refined tuning curves may be recorded or even active hearing mechanisms like those in tree crickets (Mhatre and Robert, 2013) may be revealed.

#### Frequency tuning of auditory afferents

The biophysical and neurophysiological basis for frequency tuning of the auditory afferents are not yet resolved in detail. They may depend on the opening state of the spiracles (Kostarakos et al., 2009), the properties of the tracheal tubes, and also on intrinsic properties of the sensory neurons. The 40–60 afferent neurons are linearly arranged over a distance of 300 μm in the tonotopically organized crista acustica, in which sensory neurons responding to low frequencies are located proximally and neurons responding to high frequencies are located distally (Oldfield et al., 1986). The sensory neurons are positioned right on the surface of the anterior branch of the auditory trachea while their dendrites project into a attachment cells of systematically varying size, which are linked to the lateral cuticle of the tibia (Michel, 1974). The auditory sensory neurons may be activated in a frequency-specific way by sound-induced traveling waves in the auditory trachea, mechanically stimulating the dendrites and opening mechanically gated ion channels. Frequency-specific traveling waves within the auditory trachea of bushcrickets have recently been described (Montealegre-Z et al., 2012; Udayashankar et al., 2012); the waves preferably elicit oscillations of the auditory trachea in a tonotopically arranged gradient along the crista acustica and appear to establish the tuning of the auditory afferents.

At the afferent population level, the summed activity of the auditory nerve in *G. campestris*, shows the lowest threshold for hearing to be around 50 dB SPL for sound pulses of 4.0–4.5 kHz, i.e., in the range of male calling song (Figure 3B; Nocke, 1972). The hearing threshold sharply increases toward 2 kHz, but toward higher frequencies, a secondary broad-threshold minimum at 65 dB SPL occurs for sound of 7–9 kHz. The system becomes increasingly less sensitive toward 10–12 kHz, but sensitivity subsequently increases, with the threshold dropping to 60 dB SPL at 14 kHz. Overall, in the range of 4–6 kHz the threshold curve corresponds well with the velocity response of the tympanic membrane (Figure 3A). The summed activity of the auditory nerve comprises the response of many auditory afferents, each of which has a lowest threshold of around 45 dB SPL (Esch et al., 1980; Oldfield et al., 1986). The tuning of the individual auditory afferents shows a discontinuous distribution of best frequencies, with about 75% of afferents responding to the carrier frequency of male calling song (Zaretsky and Eibl, 1978; Esch et al., 1980; Imaizumi and Pollack, 1999), and with the remaining to high frequencies that represent the male courtship song with dominant frequencies of 11–16 kHz (Libersat et al., 1994) and ultrasound sonar calls of echolocating bats above 20 kHz. This discontinuous distribution of best frequencies may reflect the frequencies of the most behaviorally relevant sounds for females, as they must respond with positive phonotaxis to the signals of conspecific males and with negative phonotaxis to the calls of predatory bats (Wyttenbach et al., 1996; Imaizumi and Pollack, 1999).

The activity of the auditory afferents is carried toward the prothoracic ganglion where their axons terminate in the anterior ventral neuropil (Eibl and Huber, 1979; Wohlers and Huber, 1985). Axonal arborizations are tonotopically arranged with afferents tuned to calling song projecting more medially, and afferents tuned to sounds of higher frequencies projecting more laterally (Imaizumi and Pollack, 2005). In *Teleogryllus oceanicus*, and likely also in *G. bimaculatus*, the bifurcating axons of afferents tuned to calling song project more posteriorly. They may connect to descending interneurons like the DN1 neurons, which forward signals in the frequency range of the calling song to the posterior thoracic ganglia (Esch et al., 1980; Wohlers and Huber, 1982; Imaizumi and Pollack, 2005). Details of auditory processing in these ganglia are, however still not well analyzed.

#### Tuning of thoracic interneurons

**Figure 4:**
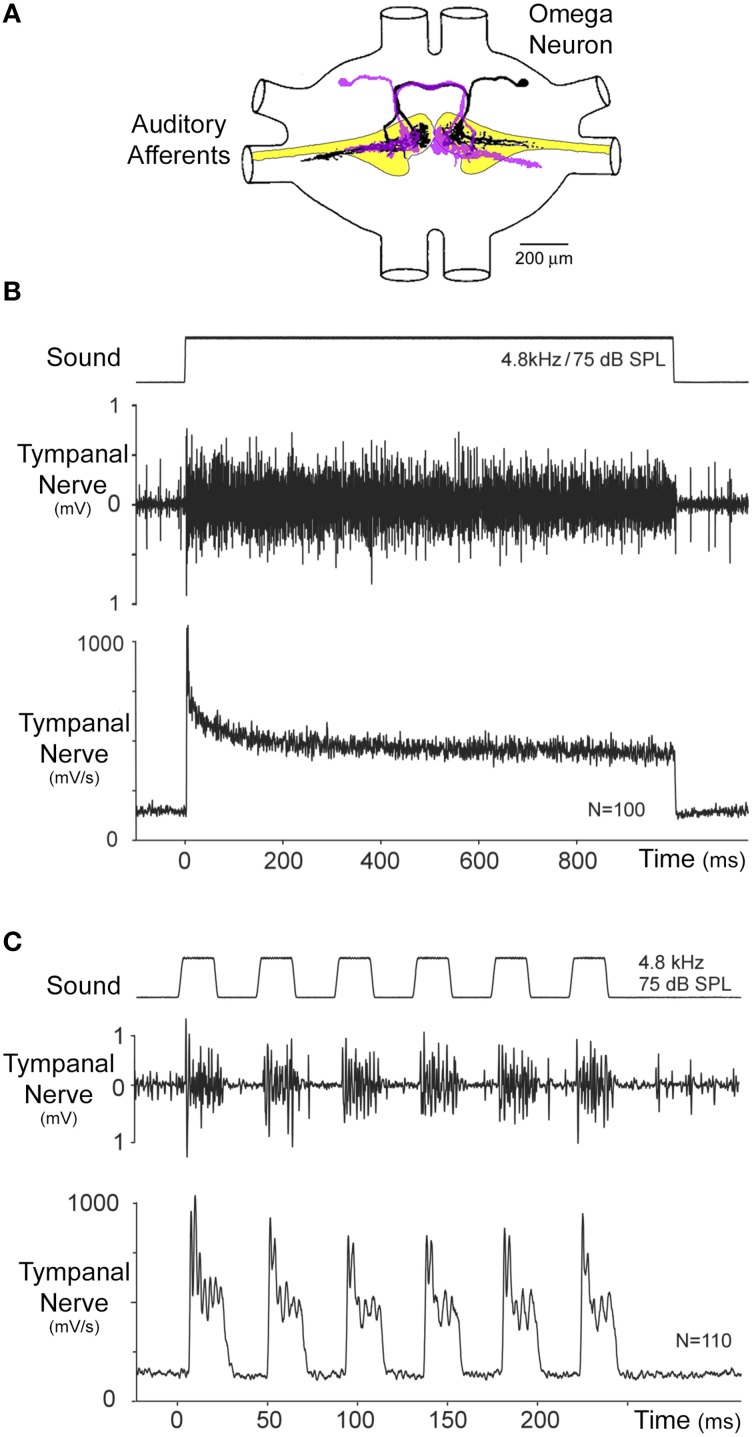
**(A)** Arrangement of auditory afferents (yellow) and the mirror image ON1 neurons in the prothoracic ganglion, redrawn from Wohlers and Huber (1985). **(B)** Phasic-tonic response of the auditory afferents as revealed by a summed recording of the tympanal nerve in the distal femur. Stimulation with a 1000 ms pulse demonstrates the phasic synchronized onset response of the auditory afferents, which adapts to a tonic activity level. The phasic response is clearly revealed by averaging the rectified neural signal. **(C)** Acoustic stimulation with the species-specific pulse pattern shows the phasic onset and representation of the pulse pattern in the summed afferent activity. Modified from Nabatiyan et al. (2003).

In the prothoracic ganglion, afferents make synaptic contact to bilateral pairs of local (ON1, ON2), descending (DN1), ascending (AN1, AN2), and T-shaped (TN1) auditory interneurons (Wohlers and Huber, 1982; Imaizumi and Pollack, 2005). The local omega shaped ON1 neurons (see Figure 4A) respond most strongly to the carrier frequency of the calling song, but also respond to the high frequency components of courtship songs or bat calls (Marsat and Pollack, 2004). The two pairs of bilateral ascending interneurons forward information from the thoracic auditory neuropil toward the brain. AN1 (Figure 3C) is tuned to the male calling song (Rheinlaender et al., 1976; Schildberger et al., 1989; Horseman and Huber, 1994). It has its lowest threshold of about 43 dB SPL at around 5 kHz, and matches the best tuning of both the posterior tympanal membrane and the auditory nerve. The threshold of AN1 increases sharply to 80 dB SPL from 2 to 5 kHz and increases gradually to 80 dB SPL from 5 to 12 kHz (Figure 3C). AN1 is the only neuron that carries information about the calling song toward the brain. Schildberger and Hörner (1988) provided an experimental proof for the close link of AN1 activity and phonotaxis. Manipulation of the AN1 spike activity by intracellular current injection in phonotactic walking crickets changed the female's walking direction and performance. AN2 and TN1, the only other interneurons with an ascending axon, are tuned to high frequencies and do not reliably copy the calling song pattern (Wohlers and Huber, 1982). The tuning to high frequency sounds may relate to courtship song or the calls of bats (Libersat et al., 1994). Therefore, high frequency signals alone are not sufficient to reliably indicate a courting male or an echolocating bat.

#### Frequency tuning of the phonotactic behavior in *G. bimaculatus*

The frequency tuning of the auditory pathway closely corresponds to the frequency tuning of female phonotactic behavior (Figure 3D). If female *G. bimaculatus* crickets walking on a trackball (Hedwig and Poulet, 2005) are exposed to 75 dB SPL calling song patterns with systematic alteration of the carrier frequency, phonotactic steering toward the sound source is strongest at 4.5–5.0 kHz. From this maximum response, phonotactic behavior decreases sharply toward 3 kHz and becomes gradually weaker toward 12 kHz. There is a good match between the frequency tuning of phonotactic behavior and the tuning of AN1, which was also established by direct comparison of AN1 activity and lateral steering (Kostarakos et al., 2008). A similar match has also been indicated for the carrier frequency and the tuning of the AN1 interneuron in *Teleogryllus commodus* (Hill, 1974).

### Neural representation of sound pulses

Patterns of sound pulses and silent intervals are a characteristic element of cricket songs. Sensory processing at the level of auditory afferents and first order interneurons may therefore be adapted to respond specifically to the temporal structure of the sounds and to represent it in patterns of neural activity.

#### Phasic-tonic responses of auditory afferents

The primary auditory neurons are scolopidial mechanoreceptors (Michel, 1974), and show phasic-tonic response characteristics (Nocke, 1972; Oldfield et al., 1986; Nabatiyan et al., 2003). They project into the prothoracic ganglion and activate first order interneurons like ON1 (Figure 4A). When stimulated with a 1000 ms sound pulse at the carrier frequency of calling song (Figure 4B), summed recordings from the auditory nerve show a salient response of the afferents to the onset of the pulse. It is best revealed by averaging the rectified (i.e., the negative signal components have been made positive) nerve recording; this procedure preserves the tonic activity component, which otherwise is lost when the signal is directly processed. The phasic onset of the auditory nerve is in the range of twice the amplitude of the subsequent tonic response. The onset response rapidly decays within 10–20 ms and then gradually to the lower level of the tonic response. Intracellular recordings from single auditory afferents showed a high spike-rate activity at the sound onset (Oldfield et al., 1986). During stimulation with a repetitive pulse pattern corresponding to the *G. bimaculatus* calling song (Figure 4C), the phasic component of the afferent activity reliably encodes the sound pulses, generating a peak response at the beginning of each pulse, even for pulse repetition rates higher than the pulse rate of the calling song (Nabatiyan et al., 2003). Thus, the phasic-tonic response properties of the population of auditory afferents allow the temporal structure of the pulse pattern to be forwarded reliably to the central nervous system. Based on the spike response of single afferents in *T. oceanicus*, Marsat and Pollack (2004) calculated the information transfer rates as bits/s transmitted by the spike patterns at different amplitude modulation frequencies. The information transfer rate of the afferents broadly represented a spectrum of amplitude-modulated sounds up to 150 Hz. Therefore, the response range of the afferents is not specifically tuned to the species-specific pulse pattern of the calling song; the filtering for the temporal pattern rather must be achieved in the central nervous system.

#### Sharpening sound onset responses by reciprocal inhibition in thoracic ON1 neurons

**Figure 5:**
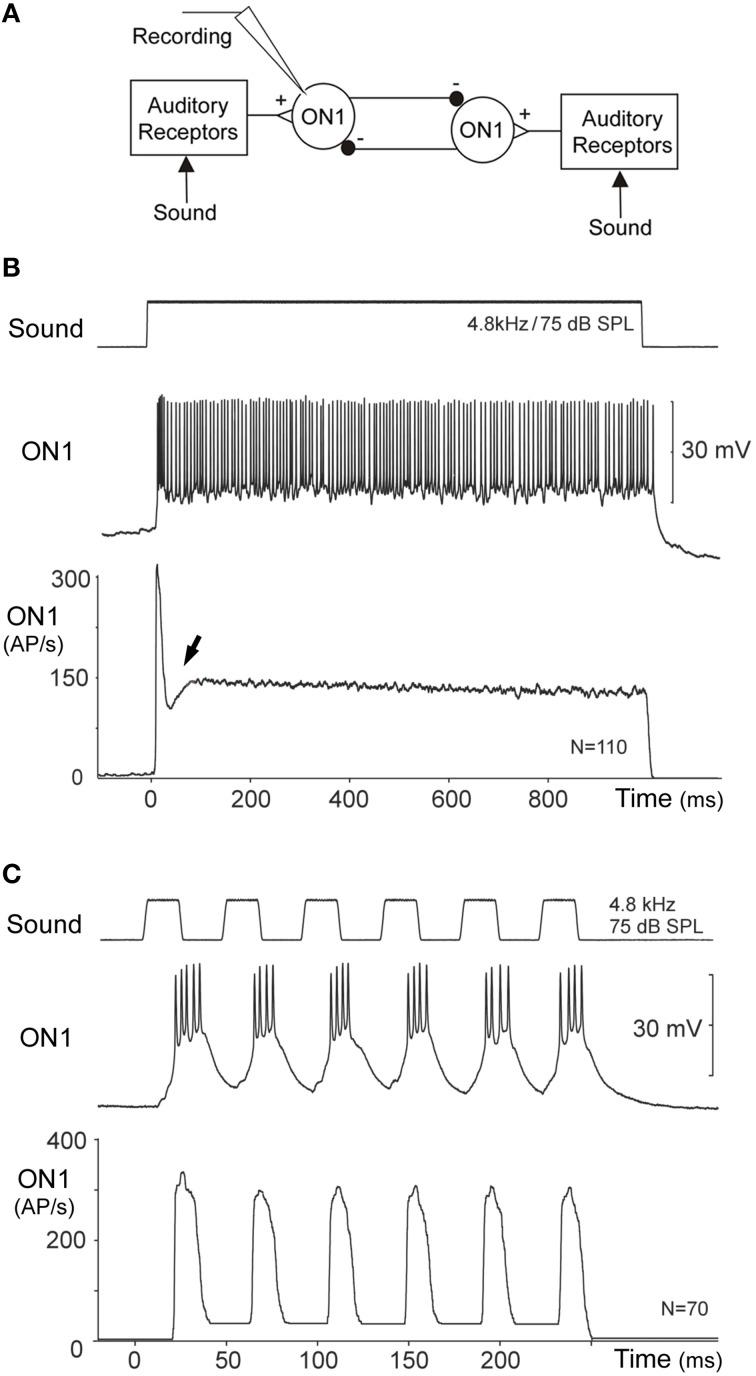
**(A)** Diagram of the reciprocal inhibitory connection between ON1 neurons. **(B)** Intracellularly recorded activity of an ON1 neuron in response to a 1000 ms acoustic stimulus presented simultaneously to both ears. Averaging the instantaneous spike rate of ON1 reveals a pronounced phasic onset response to the pulse, followed by a reduced spike rate after the onset response (arrow) with a subsequent tonic activity level. **(C)** During acoustic stimulation with the species-specific pulse pattern, ON1 activity mirrors the temporal pattern of the signal in its spike activity and the instantaneous spike rate.

The time course of the afferent response is mirrored in the response pattern of the local ON1 neurons (Figures 4A, 5A), a bilateral pair of first order interneurons (Casaday and Hoy, 1977; Popov et al., 1978; Wohlers and Huber, 1978, 1982). Each ON1 neuron receives synaptic input from the auditory afferents of the ear ipsilateral to its dendritic field while its axon projects to the contralateral side. As the auditory phasic onset response sums across all afferents tuned to the calling song (Ronacher and Römer, 1985; Pollack and Faulkes, 1998), the onset of sound pulses also leads to a pronounced phasic response in these interneurons.

The bilateral pair of ON1 neurons is coupled by reciprocal inhibition (Figure 5A; Selverston et al., 1985), which has been suggested to contribute to temporal filtering of the species-specific pulse pattern (Wiese and Eilts, 1985; Wiese and Eilts-Grimm, 1985) and may play an important role for their auditory response properties. When a 1000 ms acoustic stimulus at 75 dB SPL and 4.8 kHz is presented from the anterior, the ON1 neurons generate a transient onset response with a burst of spikes reaching instantaneous spike rates in excess of 300 AP/s. Thereafter, they rapidly stabilize to a tonic spike rate of about 150 AP/s (Figure 5B). At first sight, the time course of this response appears to be similar to the summed afferent response (Figure 4B). However, immediately following the phasic onset response of ON1, a pronounced drop in spike rate occurs, by about 50 AP/s (arrow Figure 5B), which transiently reduces the neuron's activity even below the subsequent level of tonic activity. This transient drop therefore enhances/sharpens the phasic onset activity relative to the tonic response. The fast drop in ON1 spike activity is not typical for the decline of a phasic response and it is not expected by the time course of the afferent activity. This peculiar feature may rather indicate that the neural representation of the onset of sound pulses becomes more salient at the level of ON1 neurons due to their reciprocal inhibitory connection. Upon simultaneous acoustic stimulation of both ears each ON1 neuron will be driven by afferent activity and also by the inhibition from the contralateral ON1. Due to synaptic delay and conduction time between the neurons the inhibition reaches an ON1 just after its initial peak spiking response (Selverston et al., 1985; Wiese and Eilts, 1985; see also Römer et al., 1981 for similar processing in locusts). Without substantially changing its phasic onset response, the reciprocal inhibition will have its greatest impact immediately after the onset activity and then during the tonic activity. The reciprocal inhibition, if strong enough, thereby can enhance the representation of pulse-like acoustic signals in ON1. This can be demonstrated directly: the onset response of an ON1 to sound becomes less pronounced when the contralateral hearing organ is removed and the contralateral inhibition abolished. Recording ON1, while presenting calling song like pulse patterns, reveals that the phasic onset response reliably mirrors each sound pulse with a burst of spikes (Figure 5C). Thus, following the phasic onset response of the auditory afferents, which drives the excitation of ON1, the reciprocal inhibition between the ON1 neurons can act as a mechanism that further sharpens the onset response to sound, and thereby provides an additional way to represent sequences of short sound pulses.

#### Selective attention to the louder signal

Another mechanism on a slower time scale that supports reliable coding of sound pulses is described as “selective attention” (Pollack, 1988). Continuous repetitive acoustic stimulation elicits spike activity in ON1, which causes a gradual increase in its cytosolic calcium concentration and subsequently triggers a hyperpolarizing potassium current (Sobel and Tank, 1994; Baden and Hedwig, 2007). This leads to a suppression of the ON1 neuron's spike response to low amplitude sound pulses e.g., 60 dB SPL, when they are interspersed with a louder signal of 80 dB SPL (Pollack, 1988). Due to the build-up of hyperpolarization, the response to the low intensity sound signal gradually becomes subthreshold and the ON1 spike pattern is dominated by its response to the louder signal. In a non-competitive situation with only one signal source, the mechanisms will suppress any non-specific background noise and will enhance the neural representation of the sound pattern. In a situation of competing signalers the selective attention mechanism will ensure that when a female approaches a singing male the signal from this loudest/nearest male will dominate the spike pattern of its central auditory pathway. Behavioral experiments (Simmons, 1988; Harrison et al., 2013) indicate that females orient preferentially to the calls of louder males.

### The detection of pulse periods by a delay line and coincidence detector circuit in the brain

As the simple opening and closing movements of the wings underlying sound production do not allow for a complex amplitude modulation, it is the temporal pattern of the signals that convey the male cricket's message, similar to the pulses in Morse code. Detecting the specific temporal sequence of sound pulses, i.e., the pulse period and the chirp pattern, is not achieved at the level of the auditory afferents, and so requires more complex processing in the central nervous system. Only the bilateral pair of AN1 interneurons forwards auditory signals in the range of calling song to the brain; another pair of neurons (AN2) responds to high frequency signals. AN1 is not tuned to the temporal pattern of the calling song (Wohlers and Huber, 1982; Schildberger, 1984b) and reliably responds to different temporal patterns of sound pulses, although, the spike rate of the response decreases at high pulse repetition rates. Therefore, besides some pre-filtering that occurs at the thoracic level, the final processing and selective detection of the species-specific pulse rate must occur in the brain. Furthermore, crickets in which the connectives to the brain have been severed do not show any positive or negative phonotactic responses (Pollack and Hoy, 1981).

#### Circuit structure

**Figure 6:**
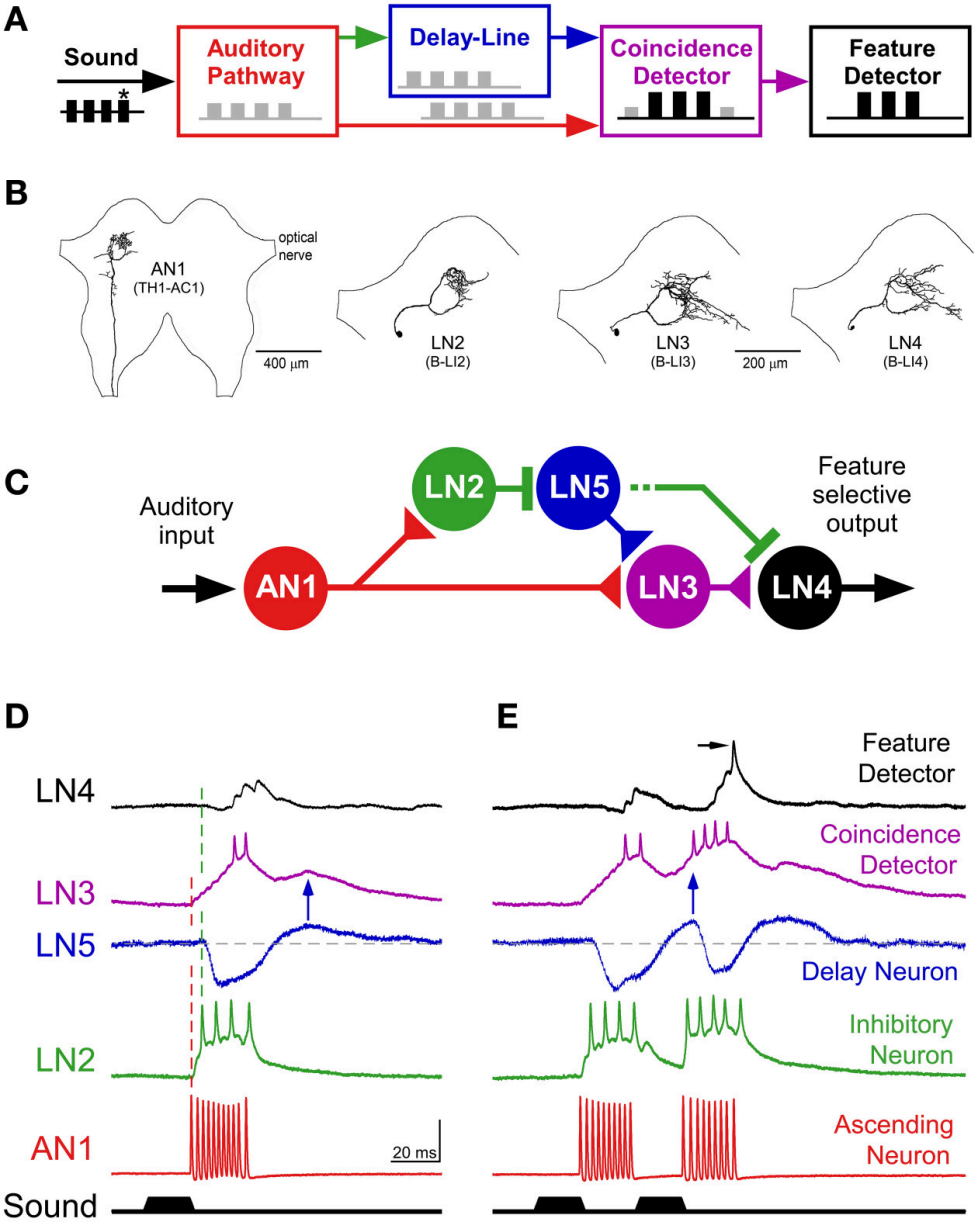
**Feature detection in the cricket brain. (A)** Delay line coincidence detector circuitry proposed for feature detection of the species-specific pulse rate, after Weber and Thorson (1989). **(B)** Axonal arborizations of AN1 and local auditory brain neurons LN2–LN4 in the anterior protocerebrum, neurons are labeled as “B-LI” neurons in Kostarakos and Hedwig (2012). **(C)** Proposed feature detecting circuitry in the brain with the ascending neuron AN1, the delay line neurons LN2 and LN5, the coincidence detecting neuron LN3 and the feature detector neuron LN4. The coincidence detector neuron integrates activity directly forwarded from AN1 and delayed activity forwarded via the delay line with the non-spiking neuron LN5. **(D)** In response to a single sound pulse, AN1 generates a burst of spikes, which directly drives the activity of the coincidence detector neuron LN3. AN1 also activates the delay line via LN2, which tightly follows the activity of AN1, and subsequently inhibits the non-spiking interneuron LN5. At the end of a sound pulse, when released from inhibition, LN5 generates a post-inhibitory rebound, which reaches its maximum amplitude after about 40 ms and elicits a delayed gradual depolarization in the coincidence detector neuron LN3 (blue arrow). In response to the first sound pulse, the response of the feature detector neuron LN4 is dominated by the inhibition via LN2, and shows minor excitation via LN3. **(E)** In response to a second sound pulse presented at the species-specific pulse interval, the coincidence detector integrates the delayed response from LN5 (blue arrow) and the direct response from AN1. The second response of the coincidence detector LN3 is boosted, now its excitatory input to LN4 overcomes the inhibition, causing the feature detector neuron to spike (arrow). **(A,C–E)** from Schöneich et al. (2015); **(B)** modified after Kostarakos and Hedwig (2012).

Several different mechanisms have been proposed to underlie the processing of the species-specific pulse rates, such as internal templates, band-pass filtering, resonant networks, and delay line coincidence detection (Hoy, 1978; Schildberger, 1984b; Weber and Thorson, 1989; Bush and Schul, 2006; Kostarakos and Hedwig, 2015). Current data provide strong support for a circuit comprising a delay line and a coincidence detector (Figure 6A; Schöneich et al., 2015), a similar circuit design was originally proposed for directional auditory processing (Jeffress, 1948) and outlined as a concept of resonant networks by Reiss (1964). Corresponding to the delay line coincidence detection concept the response to sound pulses is split into two parallel pathways. The activity in one pathway is directly forwarded to the coincidence detector whereas the activity in the parallel pathway is delayed by the species-specific pulse period before reaching the detector. Consequently, a single sound pulse will only weakly activate the coincidence detector, but when the pulse-interval of the stimulus pattern corresponds to the internal delay, the direct input, and the delayed input from the previous pulse coincide and the response of the detector will be significantly enhanced. The delay in the cricket brain cannot be achieved by axonal delay-lines as proposed for binaural processing by Jeffress (1948) and which in owls allow only microsecond delays (Carr, 1993). As processing of communication signals in the cricket brain requires delays of about 40 ms the delay rather needs to be based on an inhibitory mechanism.

The axonal projections of AN1 (Figures 3C, 6B) terminate in the frontal protocerebrum and form a ring-like arborization. A set of four local auditory interneurons (LN2–LN5) closely match this arborization pattern and form a similarly-shaped ring-like auditory neuropil in the brain (Figure 6B; Kostarakos and Hedwig, 2012); the structure of LN5 (Schöneich et al., 2015) is similar to LN2. Like AN1, these local neurons are also tuned to the carrier frequency of calling song (Schöneich et al., 2015). The response properties of these neurons together constitute a delay line coincidence detection circuit as outlined in Figure 6C. This conclusion is supported by increasing latencies for auditory processing in the circuit and very specific synaptic responses of the neurons (Schöneich et al., 2015). Together these indicate one particular flow of activity in the circuit and allow only one most parsimonious interpretation for the function of the local circuitry (Figures 6D,E), which matches a previous hypothesis on pattern recognition (Weber and Thorson, 1989).

#### Functional properties of the delay line coincidence detector circuit

A delay line coincidence detector requires two parallel pathways; a direct pathway and a delayed pathway. In the cricket brain, the direct pathway is based on the connection between AN1 and the coincidence detector neuron, LN3 (Figure 6C). The delayed pathway appears to be set up via two neurons, an inhibitory neuron, LN2, which closely follows the activity of AN1, and a non-spiking interneuron, LN5 (Schöneich et al., 2015), which is inhibited for the 20 ms duration of a single sound pulse. At the end of a sound pulse, and so release of inhibition from LN2, neuron LN5 generates an excitatory post-inhibitory rebound response which reaches its maximum about 40 ms after the end of the sound pulse, corresponding to the duration of the pulse period (Figure 6D). This post-inhibitory rebound response can also be induced experimentally by applying a hyperpolarizing current pulse, and is produced upon the offset of the current pulse, i.e., when the hyperpolarization is removed. The post-inhibitory rebound has the same amplitude for stimulus intensities in the range of 50–80 dB SPL and thus provides a mechanism for intensity-independent auditory processing, which is a fundamental property of pattern recognition processes. The rebound response is also independent of stimulus duration, in the range of 10 to about 50 ms. For a delay line coincidence detector network, a coincidence detecting neuron should only respond when the direct and delayed pathways coincide, and one of the local brain neurons, LN3 exhibits response properties characteristic of a coincidence detector. Intracellular recordings of its synaptic activity indicate that the direct input to this neuron is provided by AN1, and the delayed input, based on the post-inhibitory rebound by the non-spiking interneuron, LN5. The response of LN3 to a single sound pulse is low (Figure 6D). However, if two pulses are presented at the species-specific pulse period of about 40 ms, its synaptic input and spike activity considerably increase, by a factor of 2.3 (Figure 6E). At lower pulse rates the direct and the delayed excitation to the coincidence detector are out of sync, and at higher pulse rates AN1 spike activity does not properly represent the sound pulses (see Schöneich et al., 2015 for details). Therefore, from the properties of this network, the neural circuitry responds best to the species-specific pulse rate (Figure 6E). The final element in this circuitry is the LN4 neuron. Its spike response to single sound pulses is subthreshold (Figure 6D) but it responds with 1–2 spikes if a second pulse arrives at the right interval (Figure 6E). This neuron integrates excitatory and inhibitory inputs, and its tuning toward different temporal patterns becomes more specific than the response of the coincidence detector neuron. This is due to the inhibition that suppresses spiking responses toward single sound pulses, and allows only sound pulses with the right interval to elicit spikes. Therefore, the LN4 is selectively only activated by the species-specific pulse pattern and acts like a feature detector for calling song. The neuron shows a band-pass tuning curve in its spike activity that very closely matches the tuning of female phonotactic behavior (Kostarakos and Hedwig, 2012). The evidence demonstrates that processing of the pulse rate occurs within the local network of ring-like brain neurons, which form a close association with the arborizations of AN1. This neural network may therefore represent the filter mechanism or feature detector circuit for the pulse pattern of calling song in crickets like *G. bimaculatus*. The auditory activity of other neurons in the brain with band-pass tuning curves similar to LN4 may be a consequence of this early processing mechanism (Schildberger, 1984b; Zorovič and Hedwig, 2011).

Interestingly, the overall auditory response within the circuit i.e., the number of spikes elicited per chirp, decreases at different levels of processing from AN1 to LN4 by about 90% (Kostarakos and Hedwig, 2012). This points toward sparse coding of the stimulus pattern (Olshausen and Field, 2004), which shifts the representation of the stimulus features from a temporal code to a neuron-specific place code. Sparse coding appears to be an efficient way for simple nervous systems to ensure a robust representation of stimulus patterns.

## Processing at the chirp level: insights from pattern recognition and auditory steering

In addition to the pulse pattern of calling song, in many species of crickets (Alexander, 1962; Otte, 1992) sound pulses are grouped into chirps, which in *G. bimaculatus* are repeated at a rate of 3–4/s. In phonotactic experiments, females tolerate a range of chirp periods and respond even when chirps are presented only at a rate of 1/s (Doherty, 1985). The chirp pattern may require an additional filter mechanism on a longer time scale than the pulse repetition rate (Grobe et al., 2012). Some insights into possible mechanisms of processing at the chirp time scale can be derived from female phonotactic steering responses. When exposed to an attractive calling song signal presented from above, female crickets will have no directional cue and cannot orient toward the sound source. However, when a non-attractive sound pattern is additionally interleaved and presented from the side, a female will steer toward the non-attractive pattern. This indicates that steering is under control of the pattern recognition process and that pattern recognition and phonotactic steering are organized in a serial manner (Doherty, 1991); a pattern apparently has been recognized before the steering process is permitted.

**Figure 7:**
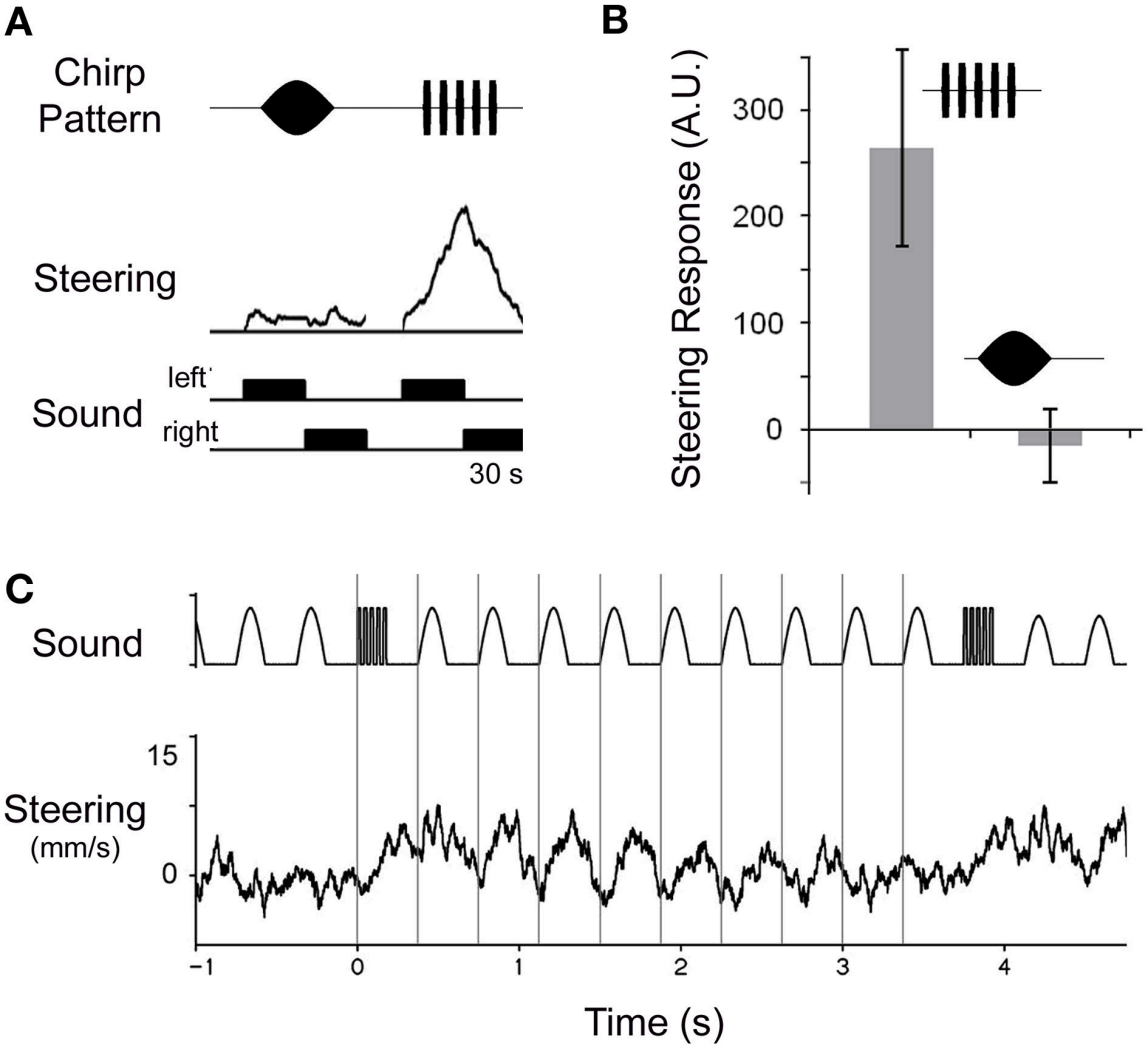
**Modulation of steering and pattern selectivity during phonotaxis. (A)** Female crickets do not steer toward an oval-shaped amplitude-modulated chirp, but respond to the normal chirp pattern when walking on a trackball. Each pattern is presented for 30 s from the left and right hand side. **(B)** Quantitative analysis of phonotactic steering in four animals demonstrates the relative attractiveness of both patterns. **(C)** When a single normal chirp pattern is interspersed into a sequence of non-attractive oval-shaped amplitude-modulated chirps; this female transiently steers toward the non-attractive pattern, indicating that processing a normal chirp modulates the subsequent processing of acoustic signals. Steering responses averaged over 15 trials.

More details can be revealed with a trackball system that measures the fast steering responses during phonotaxis. Female *G. bimaculatus* will not orient to non-attractive sounds like chirps with long sound pulses or oval-shaped amplitude-modulated sound signals presented at the natural chirp rate, whereas they readily respond toward the species-specific pulse pattern (Figures 7A,B). The females however, do steer to non-attractive chirps when these are interspersed into an ongoing calling song (Poulet and Hedwig, 2005), or in some animals even when presented just after single normal chirps, interspersed into a sequence of non-attractive chirps (Figure 7C). The readiness to orient toward non-attractive chirps gradually decays over several seconds after listening to a sequence of calling song (Poulet and Hedwig, 2005). This steering response to non-attractive patterns indicates that a modulation process on a longer time scale is initiated which modulates the auditory motor response when the species-specific pattern is processed.

Once female *G. bimaculatus* have been exposed to the calling song, they no longer evaluate the complete temporal pattern of chirps during phonotaxis, but instead steer to the first sound pulse of a chirp. They even rapidly orient toward individual sound pulses, when these are presented in a split-song paradigm, with alternating pulses on the left and the right hand side of the animals' length axis; the steering responses occur with a latency of only 55–60 ms (Hedwig and Poulet, 2004, 2005). As the recognition for the pulse rate requires at least two sound pulses (see above) the pattern recognition network can not directly provide the commands for phonotactic steering. The sparse coding at the level of the feature detector neuron LN4 (Kostarakos and Hedwig, 2012; Schöneich et al., 2015) further makes it difficult to envisage how its spike pattern might drive rapid auditory steering responses. Steering may rather involve a form of low level reactive processing which however is controlled and modulated on a longer time scale by the pattern recognition process in the brain (Poulet and Hedwig, 2005). The time-scale of this modulatory process is sufficient to explain phonotactic processing at the chirp level and could be the basis for trade-off phenomena of different song parameters as observed before (Stout et al., 1983; Doherty, 1985). A modulatory effect on phonotactic steering will be useful under natural conditions and will allow females to pursue their phonotactic approach to a calling male, even when the signal is temporally degraded due to diffraction or obstacles.

A specific neural circuitry for temporal filtering on the time scale of the chirp rate may not be required; processing at the chirp level may rather emerge from the modulatory properties of the pulse processing network. The upper limit for tolerated chirp periods could be set by the time constant of the gradually decreasing modulation effect and its lower limit may be reached when the pulse rate filter becomes ineffective, as very short chirp intervals will lead to an adaptation of the network and prevent its recovery. Whether the modulatory effect occurs within the thoracic ganglia or within the brain is unknown. The more posterior projection pattern of auditory afferents tuned to the calling song may point toward a thoracic pathway that nonetheless will be under descending control from the pattern recognition process in the brain. Processing at the level of the thoracic ganglia could provide an advantage because the auditory signals for steering could be directly forwarded to the walking motor control system with a short latency, avoiding a long loop via the brain.

## Discussion: framework and open questions

“Deciphering the brain's codes” (Konishi, 1991) is a central ongoing topic in neuroscience. In relation to sensory pattern recognition, ideas of a “single central integrator” (Barlow, 1961; Bullock, 1961), or “feature detectors” (Hoy, 1978) that represent complex sensory input at the highest level have been central, and have shaped our thinking and concepts (Martin, 1994). Experimental approaches aiming to identify such higher order feature detecting neurons and their response properties have fostered an understanding of the way that sensory systems operate when processing behaviorally relevant stimuli (Konishi, 1991). Even simple acoustic communication signals require a combination of sensory filters for a selective behavioral response. These sensory filters, such as for the amplitude, duration, or frequency of a signal, could be arranged in parallel, to finally feed into a feature detector similar to the combination-sensitive neurons in vertebrate auditory processing (Bullock, 1961; Rauschecker and Tian, 2006). Alternatively, pattern recognition may be broken down into a sequential process of autonomous stages (Barlow, 1961). The latter may be more specific, and adaptive in “simple” insect nervous systems, in which the capacity for neural processing is more restricted (Wehner, 1987). Auditory feature detection underlying cricket mate attraction points toward such a sequential solution. Otherwise, in the insect CNS and brain also multimodal neurons integrate information from different sensory pathways (Pearson et al., 1980; Schildberger, 1984a) a process which at a higher level of behavioral control may be essential for selecting and initiating adaptive motor responses (Wessnitzer and Webb, 2006).

In *G. bimaculatus* the problem of recognizing the conspecific calling song can be described as a sequence of filter processes that gradually sharpen the neuronal responses to be more selective, which eventually lead to a species-specific phonotactic motor response (Figure 2). In this sequence, only the final stage of signal processing may be regarded as a “feature detector,” whereas the lower levels provide “filtering processes.” An important functional difference between the filtering processes and the feature detector is that only the feature detector activity should be coupled to a behavioral decision that may be initiated once the detector is activated; none of the preceding filter processes should have such an impact. Several filtering steps contribute to calling song feature detection in the cricket brain, with a similar organization of sensory processing in other sensory systems.

### Processing of sound frequency

The conserved frequency tuning at different levels of the auditory pathway demonstrates that peripheral biomechanical filtering provides the essential basis for the tuning of phonotactic behavior. The frequency selectivity of female phonotactic behavior is already determined at the level of the hearing organ, and the tuning of the hearing organ defines the tuning of the auditory afferents. The detailed basis of frequency tuning in the cricket hearing organ is however not yet revealed. Auditory filter mechanisms, which tune hearing organs to the frequencies of the communication signal, are found in many other species, which depend on acoustic signals for mate attraction (grasshoppers: Meyer and Elsner, 1996, 1997), predator avoidance (moths: Schiolten et al., 1981; Fullard, 1984), and host detection (parasitic flies: Robert et al., 1992; Oshinsky and Hoy, 2002). These systems represent examples of a peripheral “matched filter,” which limits the information received by the nervous system, but simplifies the way it can be processed (Wehner, 1987).

Comparing the tuning curve of AN1 with the tuning of the auditory nerve may suggest that some additional central neural processing may sharpen the response of AN1 or rather that AN1 is selectively activated by the low frequency afferents. The data nonetheless indicate that the best mechanical response of the auditory organ drives the tuning of the majority of auditory afferents and finally the tuning of the AN1 interneuron, which matches phonotaxis (Kostarakos et al., 2008) and is crucial for phonotaxis as it provides the auditory information to the brain (Schildberger and Hörner, 1988). The response of the AN1 neuron subsequently determines the frequency tuning of brain neurons in the delay line coincidence detector network (Schöneich et al., 2015) and the tuning of the behavioral response. Like in other insect auditory systems the frequency filter in crickets is already established at the most peripheral level and provides the first filter in the calling song recognition process.

### Onset responses to sound pulses

Phasic responses of afferents and interneurons are a common feature of insect mechanoreceptive neurons (Field and Matheson, 1998). In auditory sensory neurons, they enhance the response to the onset of sound pulses (Nabatiyan et al., 2003) and are therefore suited to reliably code the timing of song patterns (Machens et al., 2003). The pool of afferent neurons with synchronously activated spike patterns (Ronacher and Römer, 1985) provides the nervous system with a robust temporal representation of regularly repeated communication signals. The prevalence of phasic responses in auditory neurons may indicate that evolution has shaped the call of male crickets into a series of regularly-repeated sound pulses in order to exploit the phasic response of the auditory afferents of females. This is in-keeping with the concept of sensory exploitation; as communication signals may evolve by the signaler exploiting pre-existing sensory biases in receivers (Ryan and Rand, 1993).

Reciprocal inhibition at the level of thoracic ON1 neurons enhances and sharpens the response to the onset of sound, and thereby is suited to especially represent short sound pulses in the activity pattern of the neurons. The dynamics of spike activity in ON1 at sound onset is in agreement with the reciprocal inhibition functioning as a temporal filter (Wiese and Eilts, 1985; Wiese and Eilts-Grimm, 1985). Based on the time constants of the transmission delay between the neurons, these authors had suggested that the tuning of the cricket auditory pathway to the calling song pattern may be due to the reciprocal inhibition, which follows the intervals of the sound pattern. However, such a filter had not yet been clearly demonstrated experimentally, as cricket auditory systems have rarely been analyzed under symmetrical stimulus conditions, like during phonotaxis when the auditory signal arrives from the front. As the strength of the inhibitory coupling may vary in different animals, the significance of this bilateral processing mechanism remains to be substantiated; it certainly is not the pattern recognition mechanism for the calling song. However, the mechanism may contribute to the enhanced information transfer, i.e., the number of bits coded by the spike patterns, in ON1 neurons for species-specific pulse rates as described in *T. oceanicus* (Marsat and Pollack, 2004). The excitatory and inhibitory inputs to ON1 neurons depend on the directionality of the ears, therefore processing at the level of the ON1 neurons may form a type of spatially selective filter for the crickets' communication signal (Marsat and Pollack, 2004). This filter mechanism would be especially important whenever the insects face a frontal signal source, such as during the approach of a singing male. As a spatially selective filter, it should play a crucial part in hyper-acute auditory orientation that allows females to steer to signal sources which are just 1–2 degree off their length axis (Schöneich and Hedwig, 2010).

The combination of the phasic-tonic response properties of the auditory afferents and the onset-enhancing mechanisms of some first order interneurons allow for an efficient neural representation of the cricket's acoustic communication pulses. Together, they can be regarded an important filtering step for the processing of calling song pulses which occurs at the thoracic level. To what degree this processing at the level of the ON1 also influences auditory activity ascending to the brain will need further elucidation.

### Detecting pulse rate—a feature detector of calling song

The recordings from brain neurons provide strong support for a circuit comprising a delay line and a coincidence detector (Schöneich et al., 2015), as outlined in a general concept of resonant network design (Reiss, 1964; Weber and Thorson, 1989; for a discussion of concepts for cricket pattern recognition such as internal templates, band-pass filtering, resonant networks see Kostarakos and Hedwig, 2015). Based on the initial filter processes, the delay line coincidence detection circuitry in the brain allows a feature detecting neuron (LN4) to selectively respond to the pulse rate of the calling song. It provides a robust description of the pulse-rate filter at a circuit and cellular level. The pulse rate tuning of the LN4 neuron matches the band-pass tuning of phonotactic behavior, as well as its frequency dependence (Kostarakos and Hedwig, 2015; Schöneich et al., 2015). The neuron therefore is a higher order neuron that can be classified as a feature detector for the cricket's calling song (Hoy, 1978).

The function of this circuitry depends on two essential processes: generation of a delay line via a post-inhibitory rebound and coincidence detection. A computational model for temporal selectivity in the acoustically communicating fish *Pollimyrus adspersus* based on a post-inhibitory rebound mechanisms, shows that temporal selectivity of the network can be tuned by the delayed time course of the post-inhibitory and by the subsequent excitatory input that coincides with the intrinsic rebound excitation (Crawford, 1997; Large and Crawford, 2002). By systematic changing the timing of the post-inhibitory rebound, this model network allows to tune output neurons to different click rates of the fish communication signal. Post-inhibitory rebound also occurs in the mouse auditory pathway where neurons in the superior olivary nucleus generate a pronounced post-inhibitory rebound underlying their selectivity for periodic low frequency amplitude modulations of sound signals (Felix et al., 2011). Post-inhibitory rebound is furthermore widely involved in precisely timed auditory processing (Koch and Grothe, 2003; Kopp-Scheinpflug et al., 2011). Delay lines and coincidence detectors covering time scales of many milliseconds are also implicated in the processing of echolocating signals in bats where they lead to topographic maps for echo delays (Suga, 1990; Kössl et al., 2014). In general they may represent a fundamental neural mechanism for processing the temporal structure of sound signals.

The feature detecting circuits are present at both sides of the protocerebrum and are coupled via local bilateral projecting neurons (Kostarakos and Hedwig, 2012), two bilateral song recognizers had been proposed by Pollack (1986). If and how the bilateral circuits interact, is not yet resolved; in acoustically communicating grasshoppers the auditory information from both sides is added in support of pattern recognition (von Helversen and von Helversen, 1995).

The field cricket *G. bimaculatus* may only need an auditory feature detecting mechanism for the pulse pattern of the calling song as rivalry and courtship signals are embedded in more complex close up encounters of mates. As the timing of sound pulses during calling and rivalry song is quite similar, the discussed filter mechanisms likely are also activated during rivalry song; whereas the high pitch courtship signals may require a different line of processing. The modulatory component in the auditory pathway, which allows for transient steering to non-attractive signals, may provide the basis for temporal filtering at the chirp level. For females, it will be sufficient to employ one neural circuit for pulse rate recognition and use a modulatory effect based on the pattern recognition process to also control responses at the time scale of the chirps.

### Control of phonotactic behavior

Female phonotaxis gradually develops and appears with sexual maturation 6–7 days after the last molt in *G. bimaculatus* (Loher et al., 1993) and 10–13 days in *G. assimilis* (Pacheco et al., 2013); it is strongly reduced after mating and upon female contact to males (Cade, 1979; Loher et al., 1993); and in *T. oceanicus* it may even depend on social experience of the larvae (Bailey and Zuk, 2009). The quality and strength of phonotaxis varies among females. Only 25–50% perform phonotaxis reliable under experimental conditions (Weber et al., 1981) and the probability that a female shows phonotaxis changes over time (Bailey, 2011). The physiological background for the maturation and variation in the strength of phonotaxis over periods of days is not yet resolved; however juvenile hormone may not play a role (Loher et al., 1992). Understanding the physiological background and moreover having neurochemical tools available to control phonotactic behavior would be a decisive advance for the neurophysiological analysis.

On a short time scale females steer to non-attractive patterns which are interspersed into calling song (Doherty, 1991; Poulet and Hedwig, 2005). This change in phonotactic behavior will be adaptive under natural conditions, when the acoustic signal quality transiently deteriorates (Forrest, 1994) and needs to be considered when interpreting behavioral data.

The location of the delay line coincidence detector circuit in the anterior protocerebrum raises a central question of how the pulse rate recognition circuit may finally initiate the phonotactic motor response. The central control mechanisms for phonotaxis is likely embedded in more general brain control architecture for insect behavior involving the central body complex (Strausfeld, 1999; Wessnitzer and Webb, 2006). Cricket auditory behavior is controlled by the circadian clock; medulla bilateral neurons project toward the neuropil dorsal to the central body and the stalk of the mushroom body (Yukizane et al., 2002). Neuropil areas in the vicinity of the central complex and the mushroom bodies are implicated in the control of singing (Huber, 1962; Otto, 1971; Hedwig, 2006; see Hoffmann et al., 2007 for grasshoppers). In flies and cockroaches the central body complex is involved in the control of walking (Strausfeld, 1999; Strauss, 2002; Bender et al., 2010). Like in some other insects in crickets it provides a compass like map for spatial orientation to polarized light (Sakura et al., 2008); yet so far we do not know to what degree it may contribute to auditory orientation during phonotaxis. The dendrites of local and descending auditory responsive brain neurons are found in the lateral accessory lobes, which generally are implicated in the control of insect motor activity and are regarded as a pre-motor region of the insect brain (Zorovič and Hedwig, 2011). Ipsilateral descending brain neurons controlling walking have also been identified in the dorsal protocerebrum (Böhm and Schildberger, 1992). However, recordings of descending brain neurons during robust phonotactic walking are still lacking; so far the reported auditory responses of such interneurons (Staudacher and Schildberger, 1998; Zorovič and Hedwig, 2013) are not sufficient to identify neural commands as required for fast and precise phonotactic steering.

The modulatory effect of pattern recognition on phonotactic steering may control auditory-motor integration at the level of thoracic networks involving posteriorly branching auditory afferents and DN1 interneurons which are tuned to the cricket calling song (Imaizumi and Pollack, 2005; Poulet and Hedwig, 2005). In such a scenario precise descending motor commands would not be required and direct reflex-like responses to auditory signals could be integrated into the walking motor pattern at the thoracic level once the modulation kicks in. How auditory steering is incorporated into the walking motor output adds another complexity to signal processing, which we just begin to understand at the behavioral level using high speed video recordings (Baden and Hedwig, 2008; Witney and Hedwig, 2011; Petrou and Webb, 2012).

Animals with specialized behavior provide model systems to analyse adapted neural processing. Particularly, insects with their “simple” nervous systems allow a detailed study of neural mechanisms at the level of identified neurons, to unravel how the system is designed to process relevant stimuli. This review focussed on data in the crickets *G. campestris* and *G. bimaculatus*. From this a comprehensive picture starts to emerge outlining the functional properties and neural basis of auditory signal processing. Pattern recognition is based on a sequence of filter mechanisms in the auditory pathway, which selectively respond to a characteristic property of the calling song and gradually sharpen the response of a neural feature detector in the brain. Based on these findings a most interesting comparative approach could reveal the filter and feature detecting mechanisms in other species, which signal with different pulse patterns for mate attraction (Alexander, 1962). Will these species use functional similar filter mechanisms and a neural circuit in the brain as a feature detector network, and in which way will the properties of the component neurons and the networks be adapted to the species-specific signals? Computational approaches based on Gabor filters have addressed this question and predict different temporal filter properties (Hennig et al., 2014; Ronacher et al., 2015). However, physiological experiments are required to reveal the actual species-specific adaptations in the neuronal mechanisms for pattern recognition that have been adapted and shaped during evolution.

## Ethic statement

Experiments in the Hedwig lab complied with the principles of Laboratory Animal Care.

## Author contributions

The author confirms being the sole contributor of this review and approved it for publication.

## Funding

Supported by the Biotechnology and Biological Sciences Research Council (BB/J01835X/1) and the Isaac Newton Trust (Trinity College, Cambridge).

### Conflict of interest statement

The author declares that the research was conducted in the absence of any commercial or financial relationships that could be construed as a potential conflict of interest.
